# Anterior hypopituitarism in a patient with amyloidosis secondary to Crohn’s disease: a case report

**DOI:** 10.1186/s13256-018-1719-7

**Published:** 2018-06-22

**Authors:** Natacha Verbeke, Nathalie Pirson, Arnaud Devresse, Raluca Furnica, Thierry Duprez, Dominique Maiter

**Affiliations:** 1 Department of Endocrinology and Nutrition, Cliniques Universitaires Saint Luc, UCL, Avenue Hippocrate 10, 1200 Woluwé-Saint-Lambert, Belgium; 2 Department of Nephrology, Cliniques Universitaires Saint Luc, UCL, Avenue Hippocrate 10, 1200 Woluwé-Saint-Lambert, Belgium; 3 Department of Neuroradiology, Cliniques Universitaires Saint Luc, UCL, Avenue Hippocrate 10, 1200 Woluwé-Saint-Lambert, Belgium

**Keywords:** Secondary amyloidosis, Crohn’s disease, Pituitary myeloid infiltration, Anterior pituitary insufficiency, Strongly hypointense pituitary gland on both T1-weighted and T2-weighted images with reduced gadolinium enhancement

## Abstract

**Background:**

Amyloid infiltration of endocrine glands has been reported, mostly in the thyroid, pancreas, adrenals, and testes, but affected patients do not frequently exhibit overt endocrine insufficiency. Here we report the case of a patient with complete anterior hypopituitarism probably due to a known systemic amyloidosis.

**Case presentation:**

Our male Caucasian patient was diagnosed with Crohn’s disease at the age of 22 years. At the age of 37, he developed secondary renal amyloidosis, which resulted in end-stage renal failure. He received a living-donor kidney transplant at the age of 57, without initial complication. Two months later, he developed extreme fatigue, weight loss, and dyspnea. A hormonal evaluation demonstrated complete anterior pituitary insufficiency. A pituitary magnetic resonance imaging was performed and showed a diffusely hypointense anterior gland on both T1-weighted and T2-weighted images with reduced gadolinium enhancement, highly suggestive of amyloid infiltration of the pituitary. Treatment was initiated with levothyroxine, orally administered hydrocortisone, and testosterone enanthate, rapidly allowing progressive marked clinical improvement and nearly complete resolution of symptoms.

**Conclusions:**

Pituitary amyloid infiltration should be considered in patients with a known systemic amyloidosis who develop symptoms of hypopituitarism and magnetic resonance imaging features compatible with protein deposits.

## Background

Amyloidosis is a single-organ or multiple-organ disease that results from extracellular deposition of insoluble fibrils causing disruption in the normal tissue architecture and eventually function impairment. Amyloid infiltration of endocrine glands has been reported in several cases of systemic amyloidosis but rarely causes endocrine dysfunction [1]. The most frequently affected gland is the thyroid [2]. Clinical involvement of the adrenal glands seems to be a rare event but diagnosis of adrenal failure is often challenging in patients with amyloidosis [3]. We report the case of a patient with Crohn’s disease and secondary amyloidosis who presented with complete anterior pituitary insufficiency. To the best of our knowledge, our patient is the third reported case of hormonally proven anterior hypopituitarism associated with systemic amyloidosis [4, 5].

## Case presentation

This male Caucasian patient was diagnosed with Crohn’s disease at the age of 22 years. At the age of 37, he developed secondary renal amyloidosis which resulted in end-stage renal failure. A renal biopsy showed the typical characteristics of amyloid deposits, avidity for Congo red and metachromatic birefringence under unidirectional polarized light**.** He received a living-donor kidney transplant at the age of 57, without initial complication. He was treated with methylprednisolone 4 mg/day, tacrolimus 13 mg/day, and mycophenolate mofetil 500 mg twice daily. Two months later, he developed extreme fatigue, weight loss, and dyspnea. There was no history of fever. A clinical examination was normal (blood pressure at 110/77 mmHg). A complete blood count revealed anemia with a normal leukocyte and platelet count, a moderate renal insufficiency, hyponatremia, and high C-reactive protein (CRP) concentrations (Table 1). Iron-binding capacity was normal and there was no clinical or biochemical evidence of diabetes insipidus.

**Table 1:** Biological and hormonal parameters of the patient at admission

Parameters	Patient	Normal values
Hemoglobin (g/dl)	9.4	13.0–18.0
Sodium (mmol/l)	132	135–145
WBC (× 10^3^/μL)	7.67	4.00–10.00
CRP (mg/L)	32.0	< 5 mg/L
Creatinine (mg/L)	1.35	0.60–1.30
Morning ACTH (ng/L)	< 2	5.0–49
Morning cortisol (nmol/l)	18.5	130–500
TSH (mU/L)	1.14	0.27–4.20
Free T4 (pmol/L)	7.2	12–22
FSH (UI/L)	0.7	1.5–12.4
LH (UI/L)	0.1	1.7–8.6
Prolactin (μg/L)	26.3	4.0–15.0
Testosterone (nmol/L)	< 0.025	9.47–28.3
IGF1 (μg/L)	66	82–271
Growth hormone (μg/L)	0.10	< 2.0
Renin (μU/L)	8.0	4–50

*ACTH* adrenocorticotropic hormone, *CRP* C-reactive protein, *FSH* follicle-stimulating hormone, *IGF1* insulin-like growth factor 1, *LH* luteinizing hormone, *T4* thyroxin, *TSH* thyroid-stimulating hormone, *WBC* white blood cells

**Fig. 1:**
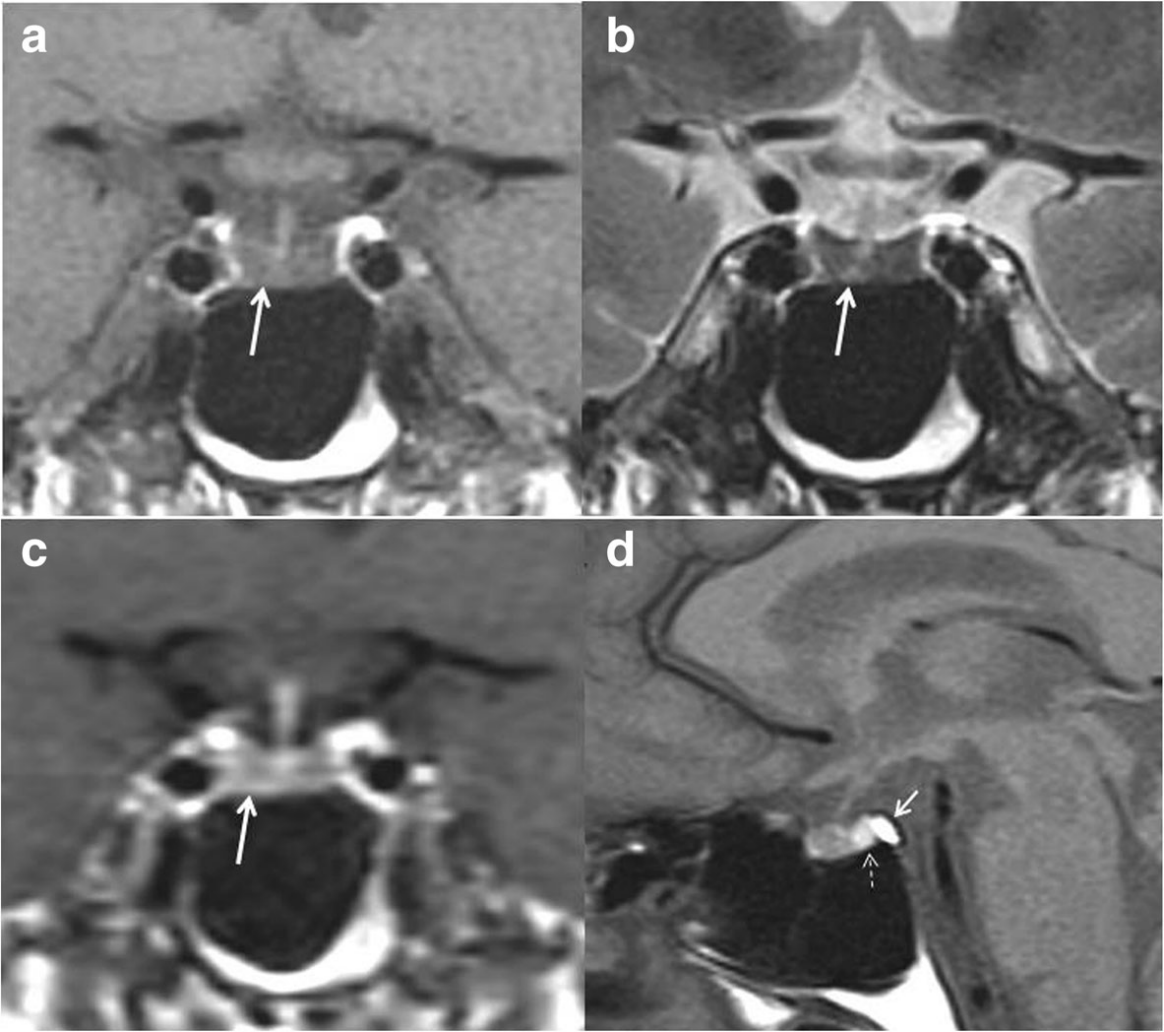
Pituitary magnetic resonance imaging performed at diagnosis. **a** Unenhanced coronal T1-weighted view showing low-intermediate signal intensity of the anterior pituitary gland (*arrow*) as compared to normal tissue. **b** Coronal T2-weighted view showing significantly decreased signal intensity of the anterior pituitary gland (*arrow*) when compared to normal. **c** Early coronal T1-weighted view after intravenous bolus contrast injection showing diffusely decreased enhancement of the pituitary gland (*arrow*). **d** Unenhanced sagittal T1-weighted view showing a small anterior pituitary gland of low-intermediate signal intensity, a normal posterior lobe displaying typical hyperintense signal (*thin arrow*), which should not be confused with the very bright signal of fatty bone marrow in the dorsum sellae (*larger arrow*). The pituitary stalk is normal

Hormonal investigations disclosed anterior pituitary insufficiency, affecting not only the corticotrope axis (which might have been related in part to ongoing glucocorticoid therapy), but also the somatotrope, gonadotrope, and thyrotrope axes, while a mild hyperprolactinemia was also observed (Table 1). A pituitary magnetic resonance imaging (MRI) was performed and showed a small anterior pituitary gland with a diffuse hypointense signal on both T1-weighted and T2-weighted images and reduced gadolinium enhancement (Fig. 1a–c). The pituitary stalk and the posterior lobe were normal (Fig. 1d). The diagnosis of pituitary amyloid infiltration was suspected and a transsphenoidal biopsy was proposed to confirm this diagnosis. However, after being clearly informed, our patient refused this invasive procedure which would not have changed the management of his disease.

Treatment was initiated with levothyroxine (87.5 μg/day), orally administered hydrocortisone (15 mg/day; to reinforce corticosteroid substitution, in addition to the low dose of methylprednisolone), and testosterone enanthate (250 mg intramuscularly/month), rapidly allowing progressive marked clinical improvement and nearly complete resolution of symptoms. Three months after hospital discharge, our patient was asymptomatic on hormonal replacement therapy, except for moderate orthostatic hypotension despite normal aldosterone and renin concentrations. This orthostatic hypotension was attributed to a possible amyloidosis-related autonomic neuropathy and was successfully treated with 9-alphafluorohydrocortisone 50 μg/day.

## Discussion and conclusions

We report the case of a patient with Crohn’s disease and secondary AA amyloidosis who presented with complete anterior pituitary insufficiency. Although no histological confirmation was obtained, the diagnosis of pituitary infiltration by amyloid was strongly suggested by the clinical context together with compatible features on MRI.

Amyloidosis is a single-organ or multiple-organ disease that results from extracellular deposition of insoluble fibrils causing disruption in the normal tissue architecture and eventually functional impairment. The current classification of amyloidosis is based on the nature of the main protein that constitutes the amyloid fibril. Primary AL amyloidosis is a systemic plasma cell disease due to deposition of proteins derived from immunoglobulin light chain fragments [6]. In contrast, secondary AA amyloidosis is characterized by deposits of acute phase reactant serum amyloid A fragments and may complicate several chronic inflammatory diseases, such as rheumatoid arthritis, inflammatory bowel disease, or chronic infections [7]. In particular, the incidence of secondary amyloidosis in patients with Crohn’s disease has been reported to be 0.5 to 8% [8].

Amyloid infiltration of endocrine glands has been reported in several cases of systemic amyloidosis but rarely causes endocrine dysfunction [1]. The most frequently affected gland is the thyroid. Thus, amyloid goiter has been described as the initial manifestation of systemic amyloidosis [2]. A recent study also showed that a significant proportion (19%) of patients with AL amyloidosis present with subclinical hypothyroidism [9]. In addition, amyloid infiltration of the thyroid gland is probably much more common than previously thought, as autopsy studies have found the presence of amyloid in the thyroid of 80% of patients with AA amyloidosis and of 50% of those with AL amyloidosis [2]*.*

Diffuse amyloid deposits may also be seen in other endocrine glands such as the pancreas, adrenals, or testes [1]. Clinical involvement of adrenal glands seems to be a rare event but diagnosis of adrenal failure is often challenging in patients with amyloidosis. Symptoms of hypotension may be erroneously attributed to autonomic neuropathy; interpretation of adrenal tests may be compromised in patients with end-stage kidney disease, and subnormal cortisol response to adrenocorticotropic hormone (ACTH) stimulation can be secondary to low level of cortisol-binding globulin caused by heavy proteinuria [3]. In such cases, ACTH and renin determinations are mandatory to exclude primary adrenal failure, as was the case in our patient.

To the best of our knowledge, our patient is the third reported case of hormonally proven anterior hypopituitarism associated with systemic amyloidosis [4, 5]. Although no histological confirmation was obtained, as in the most recently reported case [4], the diagnosis of pituitary amyloid infiltration was strongly suggested by: (i) the clinical context of a known and long-standing systemic amyloidosis; (ii) the absence of any other obvious causes that may have explained the recent clinical onset of complete anterior pituitary insufficiency (such as a pituitary tumor, hypophysitis, apoplexy, and traumatic brain injury), and (iii) above all, the features observed at pituitary MRI which were compatible with a replacement of the normal pituitary tissue with protein deposits. In fact, we observed a diffuse reduction in T1-weighted intensity and an even greater reduction in T2-weighted intensity in the whole anterior gland, a low contrast enhancement, no evidence of a tumoral or inflammatory process, and normal features of the hypothalamus, pituitary stalk, and posterior lobe. These findings are consistent with intra-glandular amyloid deposition, as previously reported by Sakai *et al.* within pituitary adenomas [10]. Moreover, other conditions which may induce a similar MRI pattern (that is, hemochromatosis, fibrosis, or previous intra-glandular hemorrhage) either could be excluded or were much less likely.

In conclusion, pituitary amyloidosis is a rare event but should be considered a possible diagnosis in any patient with a known systemic amyloidosis, who developed symptoms and signs of anterior hypopituitarism together with MRI features suggestive of diffuse protein deposits. Early treatment of secondary adrenal insufficiency is mandatory in such patients to avoid a potential life-threatening situation.
